# Reassurance and the anxious cancer patient

**DOI:** 10.1038/sj.bjc.6602077

**Published:** 2004-08-03

**Authors:** D Stark, M Kiely, A Smith, S Morley, P Selby, A House

**Affiliations:** 1 The Cancer Research UK Clinical Centre at Leeds, St James's University Hospital, Beckett Street, Leeds LS9 7TF, UK; 2 The Academic Department of Psychiatry and Behavioural Sciences, University of Leeds, 15 Hyde Terrace, Leeds LS2 9LT, UK

**Keywords:** anxiety, reassurance, remission, health anxiety

## Abstract

Many cancer patients are anxious even when disease is in remission. Anxiety about health, ‘health anxiety’, has distinct features, notably seeking medical reassurance about symptoms. Doctors may then communicate that these symptoms are not due to serious illness, a process known as ‘reassurance’. However, reassurance may inadvertently perpetuate some patients' anxiety. We aimed to observe the relation between symptoms, anxiety and reassurance in consultations with cancer patients. A total of 95 outpatients, with breast or testicular cancers in remission, completed questionnaires measuring health anxiety at study entry, then general anxiety – before a consultation, immediately afterwards, 1 week later, and before their next consultation. We examined symptoms reported and reassurance by oncologists from audio recordings of consultations, and the outcome of subjects' anxiety. The results showed that substantial health anxiety was reported by one-third of the patients. Patients with higher levels of health anxiety reported more symptoms during consultations. Reassurance was ubiquitous, but not followed by an enduring improvement in anxiety. Certain forms of reassurance predicted increased anxiety over time, particularly for subjects who were most anxious. In conclusion, health anxiety can be a problem after cancer. Reassurance may not reduce patients' anxiety. Some reassurance was counterproductive for the most anxious patients. Oncologists may need to use reassurance as a procedure, balancing risk, and benefits, and patient selection and to manage cancer patients in remission.

We have previously demonstrated that many cancer patients are highly anxious (Stark *et al*, 2002). After treatment the prevalence of morbid anxiety falls, but may not return to baseline (Noyes *et al*, 1998). There is an incomplete understanding of who remains anxious, and what oncologists can do in consultations to improve this. There may be features of anxiety about health described in settings outside cancer care, which may be observed in cancer patients, and which we can ‘translate’ to improve the management of the anxious cancer patient.

Anxiety about health may be associated with a spectrum of symptoms, such as nausea and fatigue, but a characteristic pattern of beliefs, concerns and behaviours has also been described, which is distinct from general anxiety (Forester *et al*, 1993; Lucock and Morley, 1996; Cameron *et al*, 1998). The main features of so-called ‘health anxiety’ are:

- Beliefs
  A tendency to interpret everyday bodily symptoms as indicating serious disease
- Concerns
  Intrusive worrying and preoccupation with health, to the detriment of other activities
  Fear of serious illness, such that news or events concerning illness in other people causes fear in the patient
- Behaviours
  Reassurance-seeking: seeking medical consultations, asking friends and family about bodily symptoms, or reading about illnesses (Lucock and Morley, 1996).

As cancer patients remain anxious about their health, we aim to examine whether these features are reported by patients and observed in consultations after cancer treatment (see hypothesis 1).

Surveillance is widely practiced after cancer treatment, to detect relapse and to assist patients' psychological adaptation (Working group on socio-psychological implications of follow-up, 1995). This may increase as treatment improves, and early detection expands. During clinical surveillance, patients may enquire about their symptoms. Oncologists may then seek to explain that the symptoms experienced are not due to worsening of the cancer. The process of explanation that symptoms are not due to serious disease is commonly referred to as ‘reassurance’, and has been widely studied within medical care outside oncology (McDonald *et al*, 1996; Howard and Wessely, 1996; Kathol, 1997; Lucock *et al*, 1997; Coia and Morley, 1998; Starcevic, 2001). Reassurance takes various forms, but frequently includes simple statements such as ‘not to worry’ about serious disease being present, explanation about the symptom, and radiological or other investigation (Howard and Wessely, 1996; Kathol, 1997; Coia and Morley, 1998; Price, 2000). We therefore aim to examine the frequency and types reassurance used in oncological consultations (see hypothesis 2).

When patients present with nonspecific symptoms, reassurance initially reduces general anxiety (Lucock *et al*, 1997; Conroy *et al*, 1999). Clinicians may therefore observe that their intervention has been successful. The presence of health anxiety defines a group whose general anxiety rapidly returns, which the clinician may be less aware of (Coia and Morley, 1998). Over a longer time, well-meaning reassurance may actually *maintain* general anxiety. An analogy exists with phobias, where avoiding the stimulus serves to reduce short-term anxiety, but maintains the phobia in the long term (Salkovskis and Warwick, 2001). We aim to examine whether this model is supported by the pattern of general anxiety after consultations with cancer patients (see hypothesis 3).

Health anxiety has been described in the medically well population, but after cancer the features may differ. Patients monitor their bodies for symptoms of relapse, and interpret any symptoms in that light. Therefore, cancer patients might be expected to report health anxiety as a transient phenomenon after diagnosis. Features of health anxiety, such as seeking advice about symptoms, are not necessarily unsuccessful in reducing anxiety after a major illness. Reassurance-seeking is only maladaptive if it becomes an enduring and cyclical coping pattern (Salkovskis and Warwick, 2001). Such a process might be observed during consultations for cancer patients in remission; patients report symptoms, medical reassurance follows, then a transient fall in anxiety. Anxiety rapidly returns, sometimes increased, followed by further reporting of symptoms at later consultations, perpetuating the problem. This may inform us (1) why some patients remain anxious, (2) identify a group of patients for whom reassurance is not useful and (3) indicate the potential for techniques used managing health anxiety to ‘translate’ into improved psychological outcomes.

These issues have a substantial impact upon oncology practice, affecting patient's well being and medical workload. We therefore aim to examine, in particular, several hypotheses:

1. Health anxiety will be present in cancer patients in remission
  - Health anxiety will reported by cancer patients in remission.
  - Health anxiety will predict behaviour during consultations – repeated reassurance-seeking, taking the form of enquiries about symptoms, during consultations.
2. A range of forms of reassurance will be observed in medical consultations for cancer patients in remission.
3. The pattern of anxiety after reassurance will be as predicted by observations about health anxiety in other settings.
  - Reassurance will reduce general anxiety, but only transiently.
  - Following a transient fall, general anxiety will *increase* after reassurance, particularly in subjects with high levels of health anxiety.

## METHODS

### Cohort and setting

This longitudinal observational cohort study recruited consecutive patients under outpatient review in breast or testicular tumour clinics in Leeds, UK. Eligible patients were in clinical complete remission, within 2 years of diagnosis, and undergoing follow-up at intervals of <4 months. The only exclusion criterion was inadequate literacy in English. After recruitment if there was clinical doubt that the cancer remained in remission, all data on that subject were excluded from analysis. Patients were approached prior to a medical consultation, the study discussed and written information given, but written consent was sought at their next appointment.

### Definitions

In this report, ‘health anxiety’ refers to the specific pattern of beliefs, concerns and behaviours described. ‘General anxiety’ describes a broader problem, characterised by somatic symptoms of autonomic overactivity at the immediate time of measurement. ‘Reassurance’ refers to statements communicating to patients that the symptoms are not due to serious disease, in this case that cancer is not active (Howard and Wessely, 1996; McDonald *et al*, 1996; Kathol, 1997; Lucock *et al*, 1997; Coia and Morley, 1998; Starcevic, 2001).

### Questionnaires and measures

#### The State Trait Anxiety Inventory (STAI)

The 20-item STAI-S (‘state’) questionnaire measures general anxiety at the time of completion. Items have four response categories from ‘not at all’ to ‘very much so’, giving scores from 20 to 80. In previous studies of cancer patients with anxiety disorders by standardised psychiatric criteria, mean STAI-S was 44.4, in contrast to 36 in those with normal levels of anxiety (Stark *et al*, 2002). The measure has been used for 30 years, with cancer patients, and has demonstrated factor structure and sensitivity to change (Spielberger, 1983; Fogarty *et al*, 1999).

#### The Health Anxiety Questionnaire (HAQ)

The HAQ is a 21-item questionnaire measuring beliefs, concerns and behaviours associated with health anxiety. Four response categories assess the frequency of problems over one preceding week, from ‘not at all or rarely’ to ‘most of the time’, scored 0–3. In an initial validation study of medical outpatients with unexplained symptoms, mean score was 17.4. In a lay group, the mean score was 8.6, for psychology outpatients with anxiety problems 23, and a hypochondriacal population 35 (Lucock and Morley, 1996).

We altered some items for cancer patients. Some ‘worrying about health’ is normal after cancer, so we substituted ‘how often…’ in place of the original ‘do you….’. An item referring to fear of having cancer was omitted. A question was added about interference due to worry; ‘how much has worry about your illness interfered with work, concentration or enjoyment during the last 6 months’.

### Communication during the medical consultation

The Medical Interaction Process System (MIPS) is an ‘utterance’-based analysis of verbal communication. Utterances are clauses of a sentence, ranging from single words to lengthy phrases, each of which carries a separate item of information. Each utterance is coded by the manner in which the information is exchanged, such as ‘asks open question’, or ‘interrupts’. The majority of utterances will also have a content, such as ‘related to the cancer, tests, side effects of treatment’ or ‘life-style’. These are determined by the syntax and by its context. The MIPS was derived in oncology (Ford *et al*, 2000), and has good to excellent inter-rater and rate-rerate reliability (Fallowfield *et al*, 2002). We devised and formalised adaptations to the MIPS to classify reassurance, after training from the group who developed MIPS:

*Reassurance-seeking* was defined as the patient reporting a physical symptom (Salkovskis and Warwick, 2001).

*Medical reassurance* was classified (Howard and Wessely, 1996; Kathol, 1997; Price, 2000; Starcevic, 2001):

- Either in response to a symptom raised by the patient
  - *Simple reassurance*, such as ‘this is not due to serious disease’ ‘that's ok’ ‘don't worry’.
  - *Education*. Information is given with simple assurances, for example, ‘that's due to ….’, ‘the symptom is because of …’.
  - *A plan*, For example, ‘I will take a look at that’, ‘we will do some tests’. This did not include ambivalent statements such as ‘if you have problems call us’.
- *Or s*pontaneous
  - Initiated by the doctor, not in response to a prior patients concern. Statements such as ‘you look well’, or ‘things are going well’.

DS and MK were trained by the group who developed the MIPS. DS performed the ratings, using a coding sheet developed alongside the MIPS (Ford *et al*, 2000). A random sample of 10% of interviews was selected, and rerated by DS and MK, to assess reliability (Bland and Altman, 1996).

**Figure 1:**
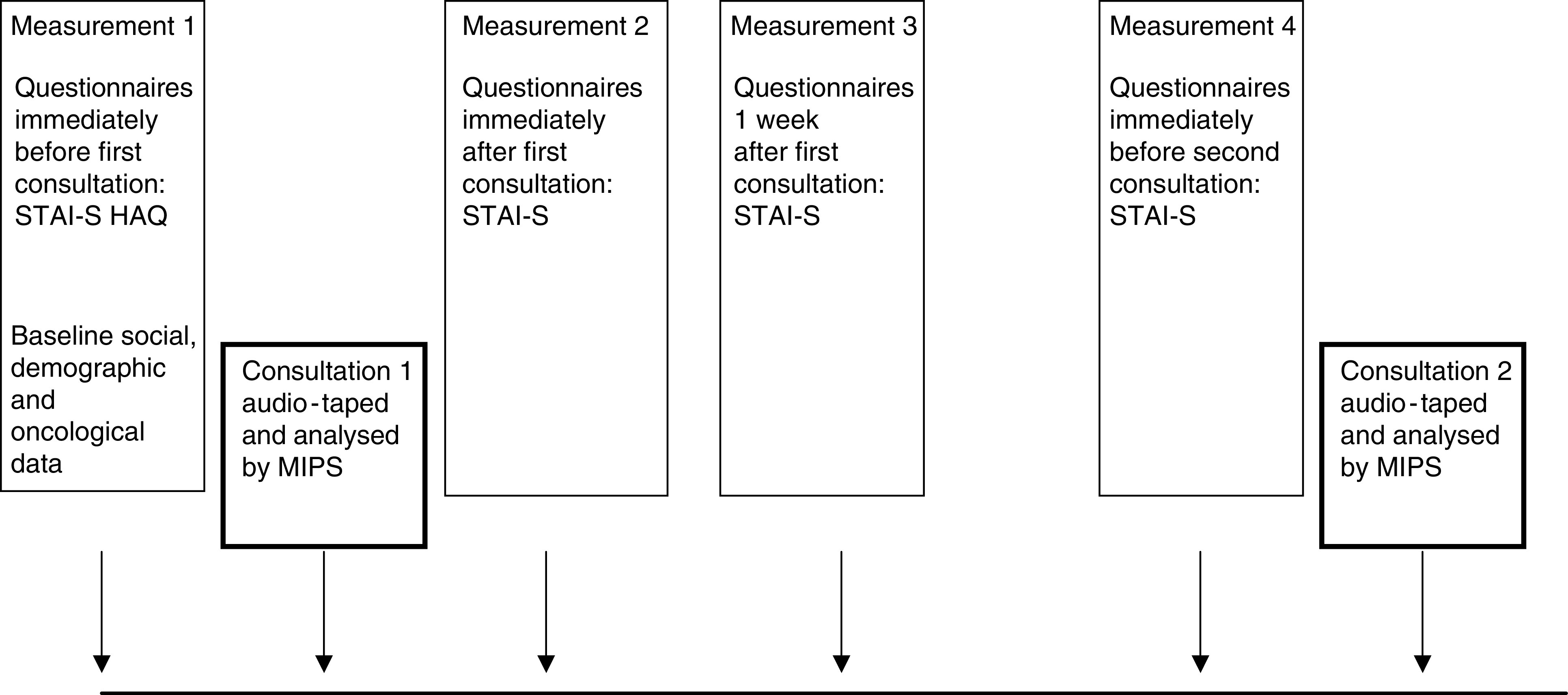
The sequence of measures.

The schedule of measures is outlined in Figure 1

. Case note review yielded age, gender and time since cancer diagnosis. Education (schooling and since school) was measured by patient report. Socioeconomic group was estimated from postcode (Stark *et al*, 2002).

### Statistical considerations

Analyses were performed with SPSS version 9. Correlations and general linear models were applied after appropriate simple transformations. Sample size was planned to permit stable multivariate analyses including potential confounding variables and planned interaction analyses. This required 10 subjects per variable, a target sample size of 90 (Altman, 1996). All variables were subject to analyses, as all were of interest *a priori*, using a general linear model. Outlying subjects were excluded from general linear models if standardised residual values were >3.3 (Tabachnick and Fidell, 1996).

The HAQ scores were skewed, so were categorised into tertiles. Previously this has delineated groups with distinct responses to reassurance (Lucock *et al*, 1997). All MIPS ratings were performed blind to other variables. Intraclass correlation coefficient was used to examine reliability of the adapted MIPS ratings, using a two-way mixed effects model (Nichols, 1998).

## RESULTS

### The Cohort, consent, missing data and attrition

A total of 160 consecutive eligible patients were offered study entry. Of these, 130 (81%) gave informed consent. A further 35 subjects gave incomplete data at 1 or more time points, due to uncertainty about relapse or patient preference. Total of 95 subjects gave data at all points. The group who gave incomplete data were more likely to have breast cancer (*χ*^2^=5.91, *P*=0.05), but similar by age and socioeconomic group (unpaired *t* tests *P*>0.25).

#### Description of the main variables (Table 1)

**Table 1:** Spearmans' rank correlations between major sociodemographic, oncological, psychological and communication variables at consultation 1

	**Descriptive data**	**Spearmans rank correlations**								
	**Mean, range, s.d.**	**Years since diagnosis**	**Full-time education**	**Anxiety**		**Number of reassurance-seeking behaviors**	**Number of medical reassurance utterances**			
				***Health anxiety***	***STAI-S***		***Simple***	***Education***	***Plan***	***Spontaneous***
Age	**45.5, 17–77, 15.9**	0.09	−0.57**	0.17	0.17	0.24*	0.22*	0.10	−0.01	−0.09
Years since diagnosis	**1.29, 0.2–2.3, 0.5**		−0.14	−0.07	−0.003	−0.05	−0.00	−0.10	−0.06	−0.14
Full-time education (years)	**12.7, 9–19, 3.1**			−0.09	−0.14	−0.22*	−0.19	−0.05	−0.09	−0.02
Health anxiety (HAQ)	**12.8, 0–54, 10.6**				0.52**	0.24*	0.23*	0.15	0.11	−0.18
General anxiety (STAI-S)	**35.5, 20–78, 11.5**					0.28**	0.31**	0.29**	0.13	−0.13
Reassurance-seeking behaviors	**1.6, 0–13, 2.1**						0.61**	0.59**	0.51**	−0.46**
*Reassurance:* simple	**2.4, 0–11, 2.5**							0.60**	0.33**	−0.53**
Educational	**2.3, 0–23, 3.5**								0.37**	−0.47**
Plan	**0.9, 0–6, 1.4**									−0.17
Spontaneous	**1.2, 0–11, 1.8**									

In bold, in the first column, are simple descriptive data; mean, range, standard deviation; STAI-S=State Trait Anxiety Inventory (state).

*N*=95. s.d.=standard deviation;

** Correlation is significant at the 0.01 level;

* significant at the 0.05 level (two-tailed).

A total of 62 subjects (65%) had testicular cancer. In all, 34% of the subjects had completed only basic school education, 41% higher education and the remaining 25% further education after age 18 years.

Health anxiety by the HAQ had good internal consistency (Cronbachs *α*=0.93) but scores were positively skewed. The highest one-third by the health anxiety score had a mean score of 24.6, reflecting health anxiety similar to psychology outpatients, and the remaining two-third had a mean score of 6.6, in keeping with a normal population. Health anxiety levels were *not* related to time since cancer diagnosis (Table 1).

Reliability of the MIPS was good. Intraclass correlation coefficients, between raters and rate-rerate, ranged from 0.69 to 0.93.

All the consultations analysed were with qualified medical staff. A total of 39 doctors contributed, but six carried out five or more consultations. The commonest symptoms reported by patients were pains, mentioned in >30 consultations. Of the 190 consultations, 97% included some form of reassurance. Simple reassurance was used in 72%, educational reassurance in 60%, plan in 38% and spontaneous reassurance in 49%. Their frequencies are given in Table 1. There was a mean of 85 utterances per consultation (range 45–717), of which reassurance constituted a mean of 7.6%.

#### Relation between the main variables (Table 1)

The subjects with testicular tumour were younger than those with breast cancer (mean 36.8 *vs* 61.9, unpaired *t*=11.2, *P*<0.001). The range of ages was wide – 16.7–72.5 for the testicular group and 44.0–77.1 for the breast cancer group. Education was very different between the two cancers; 67% of the women with breast cancer had completed basic education only, in contrast to 16% of the men with testicular cancer. There was no statistically significant difference between the two cancer types by socioeconomic status, or time since diagnosis (Mann–Whitney *U*, *P*>0.1).

The mean general anxiety at baseline was 33.7 for men and 38.9 for women (unpaired *t*=2.2, *P*=0.03). General anxiety and health anxiety are correlated, but not very strongly, suggesting they are related but distinct.

The proportion of consultations with consultants (rather than doctors in training) was 40 and 21% in subjects with breast and testicular cancer, respectively (*χ*^2^ 3.52, 2 sided *P*=0.07). Women reported more symptoms (median 5/consultation) than men (3/consultation, Mann–Whitney *U* test *P*=0.01). The number of reassurance utterances of each type per consultation was not statistically significantly different, when the two cancer types were compared (*T* tests, *P*>0.05). The 6 doctors who carried out more than 5 consultations were *not* statistically significantly different in the frequencies of types of reassurance used (Kruskal-Wallis *P*>0.15). Spontaneous reassurance was *not* more frequent when the patient had high general anxiety (Table 1).

### Health anxiety and anxiety over time

**Figure 2:**
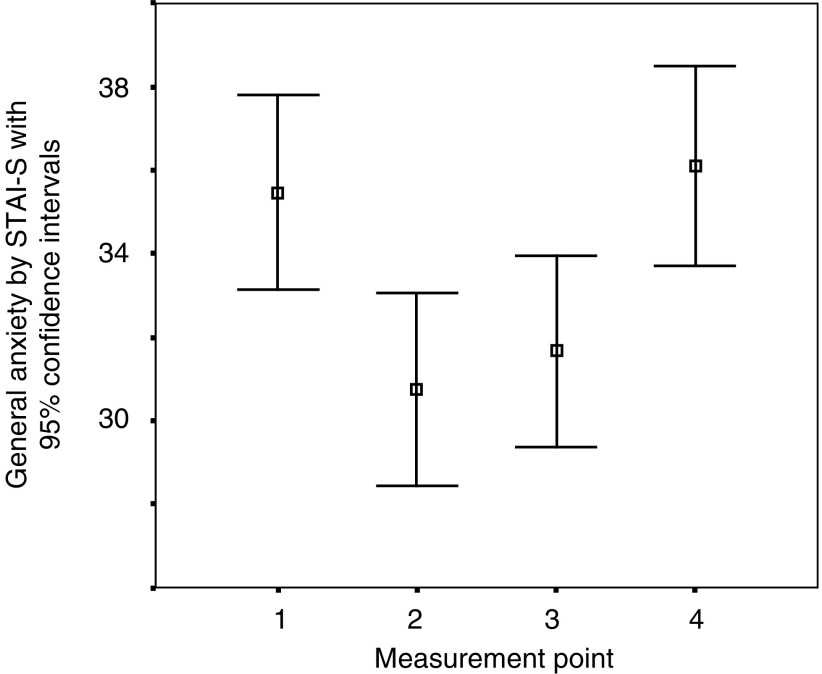
The levels of general anxiety at the 4 time points: – before the first consultation, immediately after, 1 week later and before the following consultation. *N*=95.

General anxiety measured by the STAI-S fell after the first consultation, but returned to baseline before the second consultation (Figure 2

).

The statistically significant predictors of the within-patient change in general anxiety, comparing measurement 1 to measurement 4 with a general linear model, were:- health anxiety, *B*=−0.67 (95% confidence interval −1.2 to −0.13, *f*(1,91) 3.95, *P*=0.02), and the interaction; health anxiety^*^general anxiety at baseline *B*=0.01 (95% confidence interval 0.00–0.02) *P*=0.05). General anxiety at baseline was not an independent predictor (B=0.19 (95% confidence interval –0.1–0.47), *P*=0.18). Independent of the initial level of general anxiety, higher health anxiety predicted a poorer outcome for general anxiety (Table 2

**Table 2:** Illustration of the interaction between health anxiety and general anxiety at measurement 1 in predicting the change in general anxiety from measurement 1 to measurement 4

**HAQ**	**STAI-S**	**Fall in STAI-S**
Lower (6)	Lower (25)	2.2
Higher (24)	Lower (25)	−5.3 (a rise)
Lower (6)	Higher (46.5)	7.6
Higher (24)	Higher (46.5)	3.9

Note: The values for health anxiety (HAQ, Health Anxiety Questionnaire) and general anxiety (STAI-S, State Trait Anxiety Inventory (state)) are the mean observed values for the lowest two-thirds and the highest one-third of the study distribution, respectively. The change in STAI-S is calculated from the general linear model described in the text.

).

### Health anxiety and symptoms

The two-thirds of the cohort with least health anxiety reported a mean of 1.3 symptoms/consultation, while the one-third with most health anxiety reported a mean of 2.3/consultation. Health anxiety predicted the number of symptoms reported at consultation 1 (univariate *B*=0.01 in predicting the logarithm of number of symptoms raised, *f*(1,93) 7.72, *P*=0.01). This remains of borderline significance (multivariate *B*=0.01, *f*(1,88) 3.77, *P*=0.06) when controlled for cancer type, age, education and general anxiety. Health anxiety strongly independently predicts the number of symptoms reported at consultation 2, indicating that this is a consistent behaviour (Table 3

**Table 3:** A multivariate linear regression model predicting the (logarithm of the) number of symptoms raised at the second recorded consultation, from health anxiety at measurement 1, oncological and demographic variables, and general anxiety at measurement 4

			**95% confidence interval**		
**Variable**	**Category**	***B***	**Lower**	**Upper**	***P***
Cancer type (compared to breast cancer)	Testicular cancer	−0.05	−0.25	0.16	0.65
Age		0.00	−0.01	0.00	0.34
School education, comparing to basic education	Higher	−0.27	−0.48	−0.06	0.01
	Further	−0.24	−.041	−0.07	0.01
	Overall				0.02
General anxiety at measurement point 4		−0.00	−0.01	0.00	0.65
Health Anxiety at measurement point 1		0.01	0.00	0.02	0.01

*N*=95.

Adjusted *R*^2^=0.13.

).

### Medical reassurance and the outcome of anxiety

The immediate fall in general anxiety seen after the first consultation was not related to the type or amount of reassurance observed (all *P*>0.20, univariate or multivariate analyses, data not shown). By measurement 3, 1 week later, the use of a plan as reassurance was associated with *increasing* anxiety by multivariate analysis (*f*(1,87) 7.69, *B*=−1.47, *P*=0.01).

Considering the within-patient change by the fourth point of assessment (Figure 1) spontaneous reassurance predicted *an increase* in general anxiety in univariate analysis (*B*=−1.13, 95% confidence interval –2.19 to –0.07, *P*=0.04). On multivariate analyses, spontaneous reassurance did not remain an independent predictor of the change in anxiety (*f*(1,91) 2.46, *B*=−0.78, *P*=0.12). The change in general anxiety by the following appointment was not predicted by an interaction between health anxiety and spontaneous reassurance either. The association between spontaneous reassurance and a later increase in general anxiety was therefore *not* a function of the health anxiety level.

We examined how spontaneous reassurance (during the first consultation, measured by the MIPS) was related to the within-patient changes in general anxiety at different baseline levels of general anxiety. At low baseline levels of general anxiety, the amount of spontaneous reassurance had little relation to the change in general anxiety by the fourth point of assessment, prior to the second consultation. At higher levels of general anxiety, less use of spontaneous reassurance was associated with a fall in general anxiety, a regression to the mean. However, more spontaneous reassurance was followed by a *rise* in general anxiety, in the subjects who were already the most anxious (Tables 4

**Table 4:** Association between the use of spontaneous reassurance, the level of general anxiety at measurement 1 (by STAI-S), and the within-patient change in general anxiety, comparing measurement 1 to measurment 4

		**95% confidence interval**		
**Variable**	***B***	**Lower**	**Higher**	******P******
Baseline general anxiety	0.41	0.22	0.61	0.00
Time (years) since cancer diagnosis	2.72	−0.59	6.02	0.11
Number of spontaneous reassurance behaviours	3.34	−0.65	7.33	0.10
Interaction between baseline general anxiety and number of spontaneous reassurance behaviours	−0.12	−0.24	−0.008	0.04

*N*=95.

Note: The analysis is by a general linear model. This aims to predict the change in general anxiety over time, comparing measurement 1 to measurement 4. Adjusted *R*^2^=0.20.

and 5

**Table 5:** Interaction between general anxiety at measurement 1 (measured by STAI-S), and the number of spontaneous reassurance utterances (measured by MIPS during consultation 1), in predicting the change in general anxiety comparing measurement 1 to measurement 4

**Baseline general anxiety**	**Number of spontaneous reassurance utterances**	**Increase in general anxiety**
Lower (25)	Lower (0)	4.95
Lower (25)	Higher (3)	3.93
Higher (46.5)	Lower (0)	−3.87 (a fall)
Higher (46.5)	Higher (3)	2.86

Note: The values for general anxiety (STAI-S, State Trait Anxiety Inventory (state)) and number of spontaneous reassurance utterances (MIPS, Medical Interaction Process System) are the mean observed values for the lowest two-thirds and the highest one-third of the study distribution, respectively. The change in STAI-S is calculated from the general linear model described in the text.

). This suggests that for individuals with high levels of general anxiety, spontaneous medical reassurance may be counterproductive.

## DISCUSSION

Health anxiety appears common in cancer patients in remission. About one-third of subjects reported beliefs, concerns and behaviours typical of health anxiety, and comparable to patients receiving psychological treatment. Health anxiety levels are not related to time since cancer diagnosis, suggesting that they are not simply an adaptive phase. The mean level of general anxiety in the population studied was not high for a cancer patient population; one-third had levels of general anxiety likely to be clinically significant.

Despite the problems in examining such behaviours after a cancer diagnosis, we observed several features of the consultation that are typical of those found in health anxiety. We found high levels of reporting of symptoms in consultations with cancer patients in remission. Over two consecutive consultations, health anxiety predicted more symptom reporting, which is identified in the descriptions of health anxiety as a frequent reassurance-seeking behaviour (Lucock and Morley, 1996).

Reassurance that symptoms are not sinister seemed ubiquitous. Anxiety fell after consultation whatever forms of reassurance were observed, but only transiently. In cancer care as elsewhere in medicine, reassurances that the disease is not present may not result in enduring reductions in anxiety (McDonald *et al*, 1996). After 1 week of follow-up, and longer, different forms of reassurance did not have the same impact. As hypothesised, increased anxiety over time followed some forms of reassurance. Theory suggests reassurance may worsen the medium-term outcome for anxiety if it allows avoidance of the focus of anxiety (Salkovskis and Warwick, 2001). Spontaneous reassurance, reassurance offered before the patients raised their own concerns, appeared to be followed by a worse outcome for the most anxious. This could have been because the doctors were using spontaneous reassurance when they perceived the patients as anxious, but there was little relation between the use of this form of reassurance and general anxiety levels (Table 1). Another alternative interpretation of the results would be that a higher level of symptoms results in health anxiety, and reassurance-seeking. While this cannot be entirely refuted in an observational study, it is not supported by data in other settings, which suggest it the *interpretation* by the patient, not the presence of symptoms, which is the basis of health anxiety (Coia and Morley, 1998; Salkovskis and Warwick, 2001).

We observed differences, as well as some similarities, between health anxiety in this setting and when there is no diagnosis of serious disease (Coia and Morley, 1998). Notably, a poor outcome after reassurance was not specific to subjects with high levels of health anxiety, and may be more closely related to high levels of general anxiety.

### Limitations of the study

Several aspects of the study design should be considered. The cohort included cancers that differed in gender, age, education and characteristics of the consultation. Interaction analyses have been performed for the main findings. None indicate statistically significant heterogeneity of the results by cancer type (data not shown). This design cannot examine gender and cancer type separately, which merits further study. The rate of consent was high although the rate of attrition was disappointing, but we were required to be rigorous in excluding patients when progression of the cancer was considered in doubt.

Several of the measures used were adapted for this study. This had little effect upon the quality of these measures, as the distribution, reliability and correlation analyses observed are very similar to those observed using the original HAQ in other medical outpatient groups (Lucock and Morley, 1996; Lucock *et al*, 1997). The MIPS was also altered, providing further detail of reassurance utterances that were previously grouped (Ford *et al*, 2000). These patient populations were selected for study because locally they undergo protocol-based surveillance when in remission. These results are likely to generalise to other patients undergoing similar surveillance, although this merits further study.

The timing of the measures is such that some of the general anxiety observed may be anticipatory, related to the impending appointment. Worry about illness and a belief that illness is present have previously been demonstrated to return despite reassurance, and continue in measurements taken at home over 12 months (Lucock *et al*, 1997). Therefore, the patterns of fall and rise in general anxiety observed for anxious subjects are likely to reflect the patients' experiences between appointments.This study does not include the measurement of other psychological variables, such as personality, trait anxiety or depression. Further exploration of the interplay of these variables with communication within the consultation would be interesting, as they are closely related to health anxiety (Salkovskis and Warwick, 2001). However, health anxiety has previously been demonstrated to distinguish individuals with resistance to medical reassurance, independent of levels of depression or of a personality that tends to anxiety (Lucock and Morley, 1996).

### Implications for future research

There are several questions raised that could be examined in future. The pattern of symptoms, beliefs, concerns and behaviours described may help to explain why some cancer patients remain anxious. This is an observational study, and therefore causal relationships should not be inferred from this study alone. The findings do suggest that all reassurance is not the same, but also that reassurance should be considered a specific intervention, with potential benefits, but risks for some groups of patients. It has also been suggested that reassurance should be carefully timed, and particularly that an awareness of a patient's specific concerns is helpful in providing effective reassurance (Donovan and Blake, 2000; Price, 2000; Starcevic, 2001). Spontaneous reassurance was given before patients had expressed any concerns, and therefore might be expected to be problematic.

### Conclusions and implications for clinical practice

This study is observational, so limited conclusions should be drawn for clinical practice. However, the prevalence of health anxiety observed suggests that this may be a problem after cancer. Reassurance was not able to enduringly reduce anxiety, particularly for the most anxious patients, where some particular forms of reassurance may even be counterproductive.

Oncologists may need to use reassurance as a procedure, balancing risks benefits and patient selection, to manage cancer patients in remission, and forms of communication that are effective in health anxiety could provide interventions for those who do not benefit from reassurance (Price, 2000; Warwick and Salkovskis, 2001). When patients report high levels of health anxiety, reassurance may be better avoided, in particular reassurance before the patients' concerns are elicited. If this is not helpful, for some patients, it may be necessary to replace reassurance with a careful and respectful switch of the patients' focus from a physical to a psychological basis of some symptoms and behaviours. Delivered by mental health professionals where available, with care to avoid blame and by involving patients in improving their symptoms, this can be constructive and welcome to patients (Salmon *et al*, 1999). Future work on this difficult but increasingly important area for patients and oncologists may need to focus upon the efficacy and delivery of such interventions for selected patients.
